# High Temperature Deformation Mechanisms in a DLD Nickel Superalloy

**DOI:** 10.3390/ma10050457

**Published:** 2017-04-26

**Authors:** Sean Davies, Spencer Jeffs, Robert Lancaster, Gavin Baxter

**Affiliations:** 1 Institute of Structural Materials, Swansea University, Bay Campus, Fabian Way, Swansea SA1 8EN, UK; 656258@swansea.ac.uk (S.D.); r.j.lancaster@swansea.ac.uk (R.L.); 2 Rolls-Royce plc, P.O. Box 31, Derby DE24 8BJ, UK; gavin.baxter@rolls-royce.com

**Keywords:** small punch, tensile, powder bed direct laser deposition, C263

## Abstract

The realisation of employing Additive Layer Manufacturing (ALM) technologies to produce components in the aerospace industry is significantly increasing. This can be attributed to their ability to offer the near-net shape fabrication of fully dense components with a high potential for geometrical optimisation, all of which contribute to subsequent reductions in material wastage and component weight. However, the influence of this manufacturing route on the properties of aerospace alloys must first be fully understood before being actively applied in-service. Specimens from the nickel superalloy C263 have been manufactured using Powder Bed Direct Laser Deposition (PB-DLD), each with unique post-processing conditions. These variables include two build orientations, vertical and horizontal, and two different heat treatments. The effects of build orientation and post-process heat treatments on the materials’ mechanical properties have been assessed with the Small Punch Tensile (SPT) test technique, a practical test method given the limited availability of PB-DLD consolidated material. SPT testing was also conducted on a cast C263 variant to compare with PB-DLD derivatives. At both room and elevated temperature conditions, differences in mechanical performances arose between each material variant. This was found to be instigated by microstructural variations exposed through microscopic and Energy Dispersive X-ray Spectroscopy (EDS) analysis. SPT results were also compared with available uniaxial tensile data in terms of SPT peak and yield load against uniaxial ultimate tensile and yield strength.

## 1. Introduction

With the ever-changing performance and environmental demands within the aerospace industry, there is a necessity for more advanced manufacturing methods to be employed. Additive Layer Manufacturing (ALM) is an example of these advanced manufacturing technologies, which is rapidly gaining interest within this and other industrial sectors. ALM is a process that involves the net-shape fabrication of a three-dimensional structure by fusing powders with a high-energy heat source on a layer-by-layer basis [1,2]. This method offers the ability to improve the buy-to-fly ratio by minimising material wastage through reduced need for subtractive machining and improvements to achievable geometries [3]. These advantages over conventional processing routes are attractive to the aerospace industry, with the requirement for weight savings and improved fuel consumptions. 

Powder Bed Direct Laser Deposition (PB-DLD) is an ALM technique involving the consolidation of metallic powders in discrete layers via a laser heat source within an inert atmosphere, with the aim of producing a fully dense component. A pre-determined computer-aided design (CAD) file controls the laser path on each two-dimensional layer [1,4,5]. As well as creating the scope for significant weight reductions, a CAD driven system combined with small laser diameters [4] provides the capability of creating intricate cooling systems, once thought too difficult for conventional processing. However, structures built using the PB-DLD method are sensitive to the input process variables, including laser scan speed, hatch spacing and laser power. If the combinations of these variables deliver an energy density unsuitable for the material, an abundance of anomalies such as porosity and un-melted powder particles may be present within the microstructure, and, in turn, directly influence the mechanical performance [6]. PB-DLD builds typically produce material with anisotropic properties, attributed to the re-melting of previously solidified layers, along with the direction of thermal gradients and heat dissipation, thus promoting epitaxial grain growth parallel to the build direction [4,7]. Therefore, the build orientation has a large influence on the subsequent microstructure and mechanical properties of any given component. Post processing procedures such as hot isostatic pressing (HIP) [2,7] and heat treatments can be employed to help alleviate defects and microstructural texture. Consequently, it is important to characterise the microstructure-mechanical property relationships that occur between different process variables, build orientations and post processing methods before designing and implementing a PB-DLD manufactured component envisaged for aerospace application.

Small Punch (SP) testing is a miniaturised mechanical test method, first introduced in the 1980s in the USA and Japan [8], whose early applications include remnant life assessments of steels in the power generation industry and evaluation of ductility loss in neutron irradiated materials in nuclear reactors [9]. Its key advantage comes from utilising only small volumes of material, thus saving material and allowing mechanical assessments in localised regions. Recently, the test method has proven to be a useful tool to rank the mechanical performance of materials under creep and tensile-like conditions [8,10]. In some cases, stress–strain data may be obtained for ductile materials through Small Punch Tensile (SPT) by means of finite element analysis along with sequential programming [11,12]. The technique involves the biaxial deformation of a miniature disc by a static or variable load, using a hemi-spherical indenter punch, generating load-displacement or displacement-time data through the SP tensile or creep derivatives, respectively. Given that the availability of PB-DLD material can be limited, the SP test method is a favourable option to rank their mechanical properties.

For this study, the SPT approach has been employed to assess the mechanical performance of different variants of a PB-DLD nickel superalloy, C263, with the cast alloy representing a baseline material. The materials include two build orientations subjected to either a standard heat treatment (SHT) or a higher temperature solution heat treatment (HSHT). The HSHT was primarily introduced to eradicate any microstructural anisotropy that is commonly seen in ALM built components. C263 is an age-hardened superalloy designed to have good oxidation resistance, proof and creep strength [4,13]. The alloy demonstrates good ductility in welded structures due to its low γ’ volume fraction (approximately 9.5%) [13,14,15]. This attribute is preferred in the PB-DLD process as the physical phenomena expected are comparable to those found in welding. Alloys with higher volume fractions of γ’ are susceptible to cracks in weld-like processes due to the combination of ductility losses at intermediate temperatures and residual stresses, consequently proving detrimental to the mechanical performance [15]. As C263 is an alloy commonly used for high temperature applications, SPT tests were performed at an elevated temperature in addition to those performed at room temperature (RT).

## 2. Materials and Methods

### 2.1. C263 Variants

**Figure 1:**
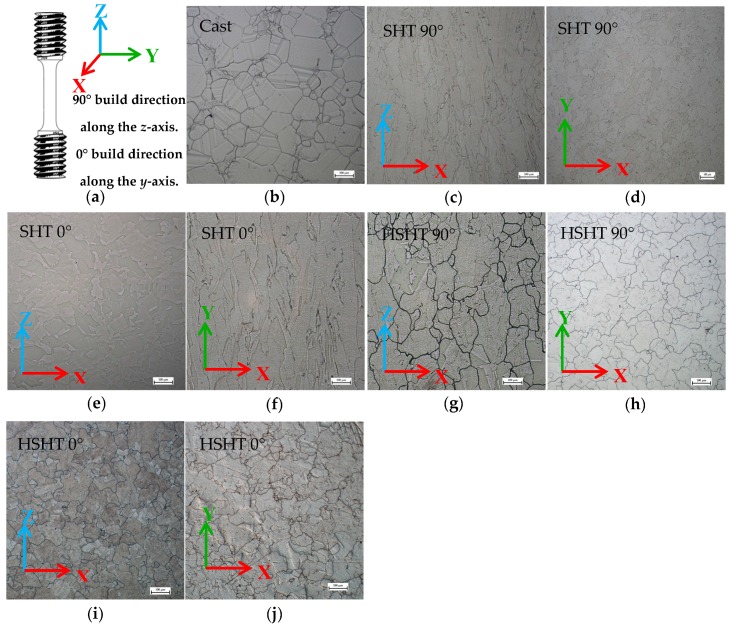
(**a**) schematic of build directions in relation to uniaxial specimens; (**b**–**j**) micrographs of C263 variant microstructures on both the *x*-*z* and *x*-*y* plane.

The microstructures of the five C263 material variants (one cast and four PB-DLD) are presented in Figure 1, with the nominal composition given in Table 1. Cast C263 (Figure 1b) displays an equiaxed microstructure with an average grain size diameter of 83 μm, obtained using the mean linear intercept method with over 250 measurements. As previously mentioned, across the four PB-DLD variants, two build orientations were tested, each perpendicular to the other—a vertical (90°) and a horizontal (0°) orientation. Figure 1a is a schematic illustrating how the build axes relate to a standard uniaxial test specimen. The SHT condition for C263 is comprised of a two-hour solution heat treatment at 1150 °C, followed by an eight-hour ageing treatment at 800 °C [4,13], whereas the HSHT condition sees the solution heat treatment temperature raised to 1275 °C. In the SHT condition, the vertically orientated specimens demonstrate a columnar microstructure parallel to the build direction (*x*-*z* plane) (Figure 1c), with a fine equiaxed morphology on the transverse plane (*x*-*y*) (Figure 1d). This grain structure has an average length of 118 μm and width of 43 μm. The microstructure of the horizontal SHT specimens seen in Figure 1e,f also display an elongated form, again parallel to the build direction; here, the grains were measured as 111 μm in length with a 52 μm width. Material built in the vertical direction that had undergone the HSHT, shown in Figure 1g,h, had an average grain length of approximately 110 μm in length with a width of 86 μm, suggesting that a columnar grain structure, although still present, is no longer as textured. In contrast, the horizontally orientated specimens after the HSHT (Figure 1i,j) display a seemingly equiaxed microstructure throughout, with an average grain size 84 μm suggesting that the HSHT has eliminated the columnar grain structure seen in Figure 1f. For all SP tests performed across the different variants, the hemispherical indenter is applied normally to the transverse plane (*x*-*y*).

### 2.2. SPT Testing

**Figure 2:**
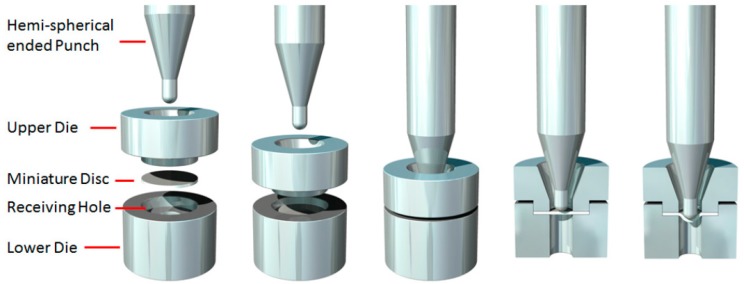
Illustration of the Small Punch test method including the assembly of the upper and lower die, miniature disc and hemi-spherical punch [9].

SPT tests were conducted using a bespoke in-house designed test jig, used to house both the SP disc specimen and the hemispherical punch, illustrated in Figure 2. All fixtures were manufactured from Nimonic-90 to allow for high temperature testing capability. The SPT jig assembly consists of an upper and lower die, designed to clamp the miniature disc specimen, where the load is applied to the top surface of the disc with a 2.5 mm diameter hemispherical punch. As the disc is deformed, the punch and fracture surface are driven through a 4 mm receiving hole. All dimensions of the jig assembly conform to the European Code of Practice (EUCoP) for Small Punch testing [17]. A threaded cylindrical support block shrouds the components, allowing the jig to be secured to a servo-actuated electric screw uniaxial test machine. The displacement rates applied were 0.3 mm·min^−1^ and 2 mm·min^−1^, reflecting the extremities of the range proposed by the EUCoP [17]. Disc displacement was measured using a standard single linear variable displacement transducer (LVDT) positioned below the disc specimen via a quartz rod, and recorded along with the instantaneous load. Elevated temperature testing at 780 °C was conducted by encasing the SPT jig within a three-zone radiant furnace. Two N-type thermocouples were used to monitor the disc temperatures to ensure they fell within ±0.25% of the desired test temperature in degrees Celsius, °C (for a 780 °C test, the tolerance is ±2.6 °C) [17].

### 2.3. Sample Preparation

PB-DLD C263 specimens were obtained by sectioning the stub ends of uniaxial test pieces that had been turned down to a ϕ9.5 mm. Cast C263 specimens were taken from ϕ9.5 mm bars extracted from a larger casted piece by Electrical Discharge Machining (EDM). The individual discs were ground down to a thickness of 500 μm ± 5 μm using incrementally finer silicon carbide abrasive papers, finishing with 1200 grit grade, in accordance with the EUCoP [17]. 

### 2.4. Microscopy

Polished specimens were etched with a swab at room temperature using Kallings 2 reagent (5 g CuCl_2_ + 100 mL HCl + 100 mL ethanol) as per ASTM E407 [18], and examined using a Reichert Jung MeF3 optical microscope (New York, NY, USA). Fractography and Energy Dispersive X-ray Spectroscopy (EDS) analysis was performed on a Hitachi-SU3500 Scanning Electron Microscope (SEM) (Krefeld, Germany).

## 3. Results

### 3.1. Small Punch Test Results

**Figure 3:**
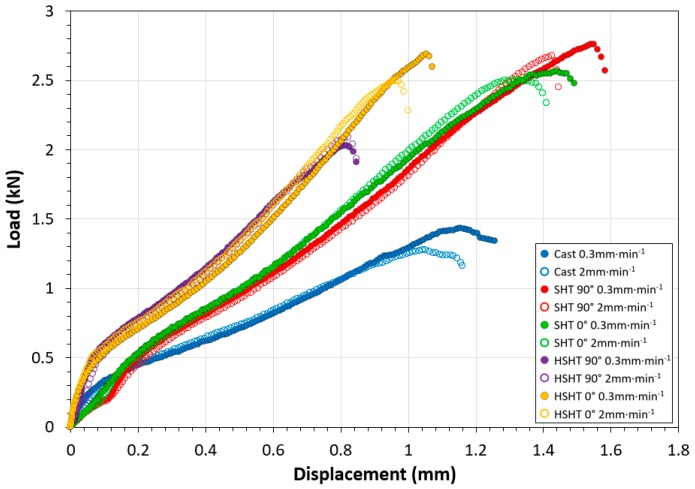
Collation of SPT load-displacement data for all material variants and displacement rates at RT.

SPT results at RT are given in Figure 3. Upon first inspection of the RT load-displacement curves, there is a distinct difference between material types, although the curve morphologies themselves are comparable to that found in a traditional SP test for a ductile material [19]. Cast C263 showed the weakest response to deformation, with a maximum load of 1.45 kN achieved by the specimen tested at the slower displacement rate. However, the PB-DLD specimens subjected to the SHT exhibited much higher peak loads and superior ductility, with a peak load of 2.80 kN at a displacement of 1.55 mm in the vertical orientation, followed by the horizontal SHT specimens, which achieved a peak load of 2.61 kN at 1.45 mm. There appeared to be very little anisotropy between build orientations for the SHT PB-DLD specimens tested, in terms of SP response. In the HSHT condition, the load accumulated per unit of displacement is much higher in both build orientations, suggesting a more brittle or hardened mechanical response. The peak loads reached by each HSHT orientation was 2.10 kN at 0.81 mm for the vertically built specimens and 2.72 kN at 1.06 mm for the horizontal type, and this difference between the two HSHT build orientations suggests that there is an element of anisotropy present.

**Figure 4:**
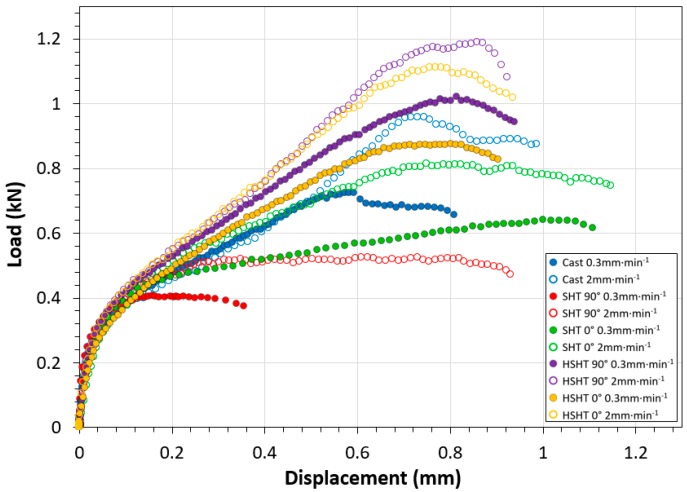
Collation of SPT load-displacement data for all material variants and displacement rates at 780 °C.

SPT results at 780 °C, displayed in Figure 4, present a significant difference in findings to those found at RT, not only in terms of curve morphology, but also in relation to the rankings of the five C263 derivatives. Firstly, there is now a prominent influence of displacement rate, with an increase in peak load associated with a faster displacement rate, similar to that observed in uniaxial tensile testing [20]. At elevated temperature, the vertically orientated SHT specimens are now the weakest performers at the higher 2.0 mm·min^−1^ displacement rate with a peak load of 0.53 kN at 0.72 mm, followed by the horizontally orientated SHT specimens with a peak load of 0.82 kN at 0.75 mm, and the cast C263 material, no longer the weakest variant, attained a maximum load of 0.97 kN at 0.74 mm. The strongest response was exhibited by the HSHT specimens, with peak loads reaching 1.20 kN at 0.85 mm for the vertically built specimen and 1.11 kN at 0.76 mm for the horizontally orientated disc. The similarities between the two build orientations in the HSHT variants suggest that the directional dependence seen in the RT tests may no longer be as significant. 

To quantify the properties that can be derived from SP tests, such as ultimate tensile strength (UTS) and yield stress, it is important to consider that the results are not immediately comparable to those found in a traditional uniaxial test due to the transient stress state. While peak load and its corresponding displacement are easily obtained from the test data, determining the yield load and displacement on an SP load-displacement curve requires more thought and has been previously discussed by several sources [8,21]. Much research has adopted the “two secants method”, which is the widely preferred approach that uses a bi-linear fit on the curve up to a point where displacement is equal to the disc thickness (0.5 mm). By minimising the error between the bi-linear function and the load-displacement curve, an intersection point can be obtained. The projection of this intersection on the load-displacement curve is recognised as the SP yield load [8,17]. 

**Figure 5:**
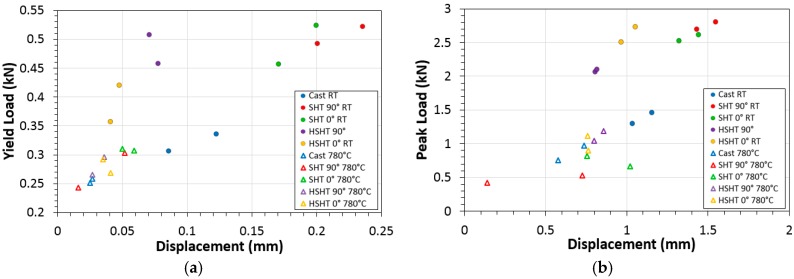
Summary of SPT data at RT and 780 °C including: (**a**) peak load–peak load displacement; (**b**) yield load–yield load displacement.

The peak load–peak load displacement values achieved in all SP tests are displayed in Figure 5a, clearly illustrating that by exposing the materials to a raised temperature condition, the mechanical performance across all variants is reduced. The elevated temperature has greatest influence on the SHT PB-DLD specimens, whilst, even though peak loads have fallen considerably, ductility does not reduce by the same extent in the HSHT specimens. The overall ranking of the variants by peak load is altered by the introduction of temperature with the cast material performance now close to or exceeding that of the SHT specimens. Figure 5b displays the calculated yield load-yield load displacement values for all tests, with the pattern akin to that of the peak load values. Interestingly, at 780 °C, the calculated SP yield values are all within 50 N of one another, suggesting the yielding mechanism is similar in all C263 variants at elevated temperature. 

### 3.2. Uniaxial Comparisons

While for a standard SPT test, only the load and displacement values are recorded, empirical correlations can be employed in order to determine an effective UTS and effective yield stress [8]. The biaxiality of the SP test as well as specimen geometry means traditional methods of converting load to stress cannot be employed. These effective stresses are calculated using the following equations:

(1) $$\sigma_{y}=\;\alpha_{1}\frac{F_{e}}{h_{0}^{2}}+\;\alpha_{2,}$$

(2) $$\sigma_{UTS}=\;\beta \prime_{1}\frac{F_{m}}{h_{0}^{2}}+\;\beta \prime_{2},$$

where $F_{e}$, $F_{m}$ and $h_{0}$ are the yield point load, peak load and specimen thickness, respectively. The parameters $\alpha_{1}$,$\;\alpha_{2}$,$\;\beta \prime_{1}$ and $\beta \prime_{2}$ are defined as correlation factors, derived by directly equating known uniaxial stress data against corresponding loads for a series of materials and temperature conditions. Garcia et al. [22] performed similar calculations and found the correlation factors for steel variations were $\sigma_{y}=\;0.476\frac{F_{e}}{{h_{0}}^{2}}$ and $\sigma_{UTS}=0.065\frac{F_{m}}{{h_{0}}^{2}}+268.8$. Nevertheless, a range of values have been reported for these parameters [17,23,24].

**Figure 6:**
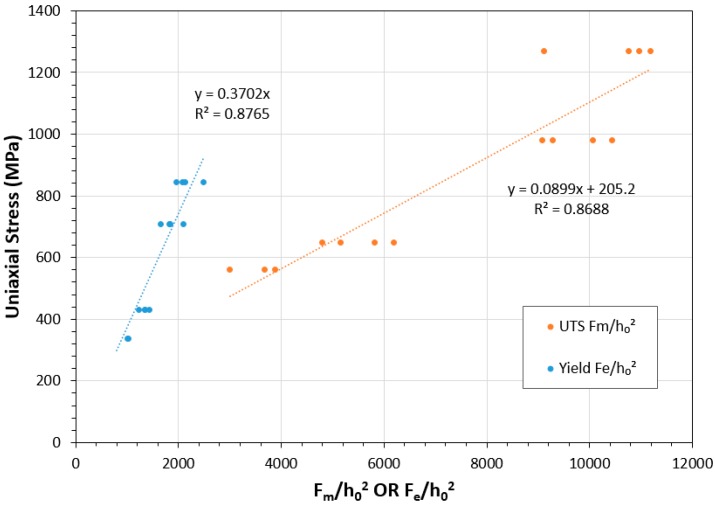
Relationship between Yield Stress vs. *F_e_*/*h*_0_^2^ and Ultimate Tensile Strength vs. *F_m_*/*h*_0_^2^ for C263 variants where uniaxial properties are known.

Figure 6 shows the relationships between yield stress and *F_e_*/*h*_0_^2^ as well as ultimate tensile strength and *F_m_*/*h*_0_^2^ for the C263 variants where the uniaxial properties were known. The uniaxial properties of $\sigma_{y}$ and $\sigma_{UTS}$ were taken from engineering design data for cast C263 at RT and 780 °C and the properties for the SHT PB-DLD variants at RT were extracted from a previous study undertaken by Vilaro et al. [4]. Here, the authors reveal the effects of build orientation on microstructure and uniaxial tensile properties for PB-DLD built C263 subjected to the same post-build heat treatment conditions used in this research.

For both the yield and UTS correlations, the R^2^ is determined to be >0.85 and the coefficients are calculated as those shown in Equations (3) and (4). The high R^2^ values determined indicate a strong relationship between the SP load and uniaxial stresses for C263 material and its variations:

(3) $$\sigma_{y}=\;0.3702\frac{F_{e}}{h_{0}^{2}},$$

(4) $$\sigma_{UTS}=\;0.0899\frac{F_{m}}{h_{0}^{2}}+205.2.$$

Through applying these equations to the load values determined for the specimens where the uniaxial data was unknown, a ranking in terms of effective yield and UTS for each of the C263 PB-DLD variants may now be completed. Table 2 shows the loads determined from the SPT tests and the calculated uniaxial properties based on Equations (3) and (4). Unfortunately, there was no uniaxial data available for the HSHT variants. A level of anisotropy is observed in the SP tests of the SHT specimens, whereas it is seen to be more prominent in the known uniaxial results. This is likely to be attributed to the biaxial nature of the SP tests, where multiple orientations of the microstructure are subjected to stress, rather than the single loading axis in uniaxial testing.

In relation to the ranking of the mechanical performance of the C263 variants, the correlated uniaxial results of course exhibit the same trend as determined by the SP results. The potential to analyse the correlated uniaxial data, in terms of a UTS, is something that could be of relevance to the industry, and while at RT the SHT variants show the greatest strength, a 60% debit at 780 °C could prove a challenge. While the HSHT samples are ranked highest at 780 °C, losing 40% of its strength from RT, it is the cast material that retains the largest proportion of its strength at elevated temperature, only seeing a 25% debit in UTS. Microstructural, fractographic and EDS analysis was carried out on all samples to try and understand these changes seen in performance.

### 3.3. Small Punch Fractography

**Figure 7:**
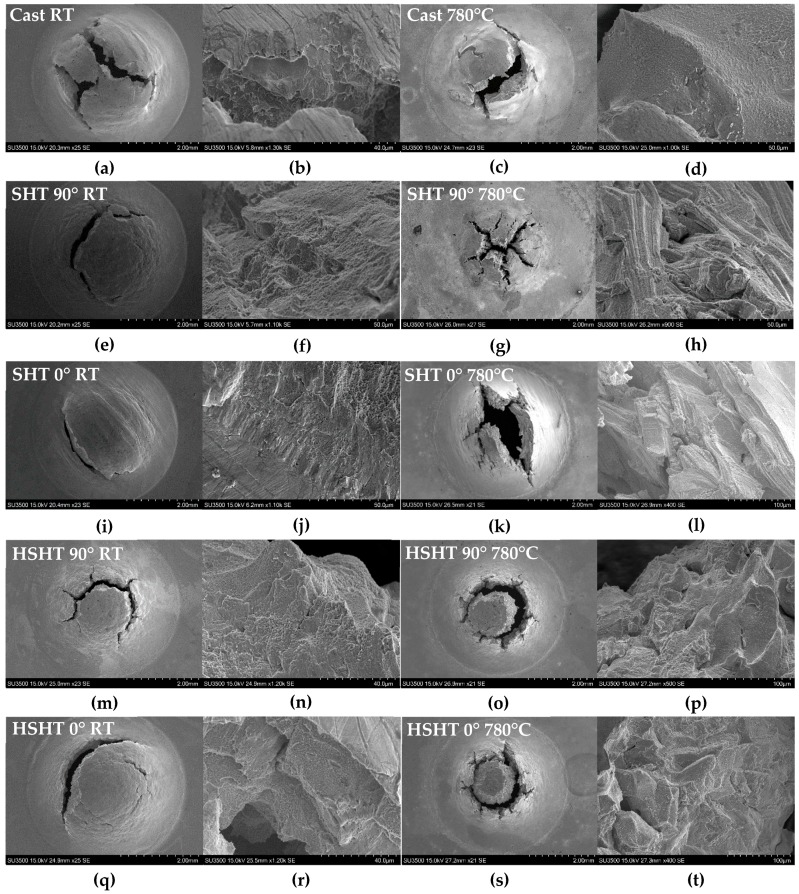
Macroscopic and microscopic micrographs of SPT disc fracture surfaces: (**a**,**b**) cast RT; (**c**,**d**) cast 780 °C; (**e**,**f**) SHT 90° RT; (**g**,**h**) SHT 90° 780 °C; (**i**,**j**) SHT 0° RT; (**k**,**l**) SHT 0° 780 °C; (**m**,**n**) HSHT 90° RT; (**o**,**p**) HSHT 90° 780 °C; (**q**,**r**) HSHT 0° RT; (**s**,**t**) HSHT 0° 780 °C.

The SP fracture surfaces depicted in Figure 7 identify key differences in deformation behaviour between each C263 variant across both temperature regimes. Cast C263 tested at RT (Figure 6b) reveals faceted features on the crack surface, with this cleavage-type fracture suggesting the material failed in a relatively brittle fashion [12,25,26,27]. At 780 °C (Figure 6d), the fracture surface was not dissimilar, showing facet-like features indicating a moderately swift rupture, highlighting the microstructural stability of the material at the two temperatures, as was identified in the SPT results and uniaxial correlations.

Viewing the macroscopic fracture surfaces of both PB-DLD SHT build orientations at RT, (Figure 7e,i) a dominant circumferential crack is visible, considered evidence of a ductile failure [26], clearly in contrast with that observed in the cast specimens. The build directions show clear anisotropy between one another, with the SHT horizontal fracture directionally aligned to the epitaxial grain growth, conflicting that seen in the SP responses, which are almost identical for both orientations. At higher magnifications (Figure 7f,j), the abundance of micro-void formation leaving a dimpled crack surface is revealed. Such fractographic features suggest the occurrence of ductile tearing during disc rupture [25,26,27]. In the elevated temperature condition, however, there is a vast difference in fracture surface topography compared to RT. Figure 7h exhibits the intergranular fracture surface of the vertically orientated SHT PB-DLD specimen at 780 °C, the grain structure been unveiled, and it shows the presence of a banded substructure. The SHT horizontal PB-DLD micrograph in Figure 7l displays evidence of an intergranular failure, as the columnar grain structure orientated transverse to the loading axis is exposed. The fact that an intergranular failure is dominant in this condition suggests that the grain boundaries are embrittled in this elevated temperature condition, thus leading to the dramatic reduction in peak load and effective UTS calculated previously [25,27].

In the HSHT PB-DLD discs (Figure 7m–t), there is circumferential cracking present at both temperatures that is coupled with evidence of radial cracking. Macroscopically, there appears to be little anisotropy, particularly in the 780 °C specimens. At RT (Figure 7n,r), micro-void formation is present, suggesting a more ductile failure [25,26,27], although not as prominent as in SHT specimens. At 780 °C, the cracking in the HSHT specimens (Figure 7p,t) transitioned towards a brittle intergranular failure mode, with large cleavage facets [25,27]. It is also apparent that the substructure highlighted in Figure 7h is not present at elevated temperatures in the HSHT condition. Overall, the fracture surfaces appear to be well related to the differences in strength identified at the two test temperatures.

### 3.4. Energy Dispersive X-ray Spectroscopy Analysis

**Figure 8:**
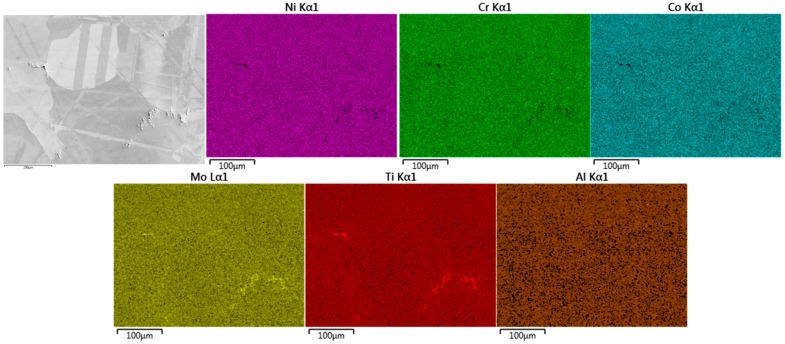
EDS analysis of cast C263 microstructure.

Energy Dispersive X-ray Spectroscopy was conducted on all material variants to understand how the differing process routes affect the dispersion of alloying elements and distribution of superalloy phases. For cast C263 (Figure 8), dispersions of carbides have been identified predominantly along grain boundaries, with occasional triple-point boundaries containing large blocky carbide precipitates. These phases have been recognised due to the increased abundance of carbide forming elements such as Mo and Ti [16], as well as a depletion of Ni, Cr and Co. These morphologies and elements detected suggest the carbides along the grain boundaries are M_6_C type, whereas the large blocky formation are MC carbides [28,29].

**Figure 9:**
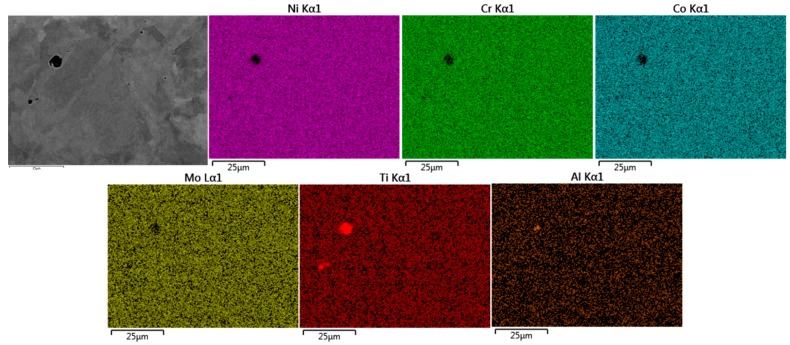
EDS analysis of PB-DLD SHT C263 microstructure.

The SHT material shown in Figure 9 appears to contain features that could prove detrimental to mechanical performance, such as porosity, a common feature within PB-DLD builds, and points of high concentrations in γ’-forming elements, such as Al and Ti [16], with these regions in Ni, Cr, Co and Mo. At the resolution available within the scope of this study, there is no clear indication of regular carbide formation in this material. At elevated temperatures, it is these elemental rich regions that are thought to contribute to the large detriment in material properties. 

**Figure 10:**
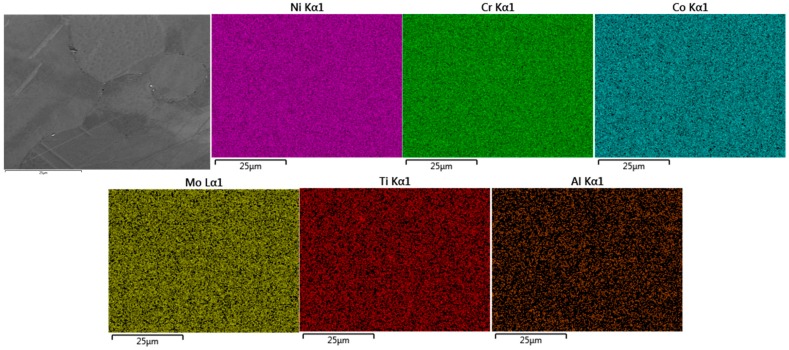
EDS analysis of PB-DLD HSHT C263 microstructure.

In contrast to the SHT PB-DLD material, the HSHT materials possess some level of finely dispersed carbides within intergranular regions, indicated by increases in weight percentages of Ti, Mo and Co (Figure 10), indicating the presence of M_6_C carbides [28]. This distribution is more comparable to that found in the cast C263 type. There is also evidence of larger morphology carbides suspended in intergranular zones, suspected to be MC carbides [29], although for a thorough characterisation of these phases, higher resolution imaging would be required. 

## 4. Discussion

### 4.1. Room Temperature Small Punch Testing

Reflecting on the microstructural images displayed in Figure 1, it is clear that the grain boundary densities in the SHT PB-DLD material in the two build orientations (Figure 1c–f) are far greater amongst these derivatives compared to the other variants. The Hall-Petch relationship on grain boundary strengthening outlines how the mechanical performance of a material is sensitive to grain size [30,31]. Therefore, reducing the grain size or increasing the number of grain boundaries would suggest an improvement in tensile properties as dislocation movement through a material is more restricted, given the difficulty in traversing to an adjacent grain that contains a large crystallographic misorientation [31]. As such, it is suggested that the higher grain boundary density at RT in the SHT PB-DLD variants is the main contributing factor allowing for an increased load capability.

HSHT PB-DLD builds demonstrated comparable strength to the SHT material, with a significant reduction in accumulated displacement suggesting an element of hardening. The EDS results showed that SHT material contained large congregations of γ’-forming elements that are not present in the HSHT material. It is assumed that, by forming as segregates, these elements are not contributing to the precipitation of γ’ during the ageing treatment; therefore, the alloy deviates from the design volume-fraction, size and morphology of γ’ precipitates, all of which directly affect mechanical response [16,32]. The HSHT material does not visibly contain these segregates, suggesting that the heat treatment has fully solutionised these elements, allowing them to contribute to the forming of γ’ precipitates, increasing the γ’ volume fraction, resulting in an increased yield and UTS at the expense of a reduction in elongation or ductility [33]. Nevertheless, this increase in strength is compromised by the material’s large grain size, subsequently creating similarities in peak loads to the SHT material at RT. 

Under the biaxial loading condition of the SPT test, the contrasting build orientations of PB-DLD C263 do not show as significant a level of anisotropy as seen in uniaxial tensile testing [4], although the macroscopic fracture surfaces highlight the textured failure mechanisms. This anisotropy is still somewhat present between the HSHT build orientations, with similar load vs. displacement traces except the HSHT 90° failures occurring at a lower level of displacement, and the fracture morphology in the HSHT 0° material aligned to the slight texture revealed in the microstructural investigations.

### 4.2. High Temperature Small Punch Testing

Fractographic analysis of the 780 °C SP tests revealed intergranular cracking to be the dominant form of propagation, suggesting results were highly grain size and grain boundary dependent. In the SHT material, a banded substructure formed due to high temperature testing, thus influencing the cracking behaviour. Consequently, its seems the SHT has not completely solutionised the dendritic structure from the as-built condition, which forms as a result of multiple constituents and rapid solidification rates [34,35], and remnants of this original structure are still present, and prove to be detrimental to the mechanical response at elevated temperatures.

Since an intergranular fracture type was dominant in all C263 variants at 780 °C, this factor would have contributed in ranking the HSHT PB-DLD with the highest peak loads. The largest grain size was found in the HSHT material, more so in the vertical orientation, and thus fewer grain boundaries for cracks to propagate. Additionally, an increase in solution heat treatment temperatures, prior to ageing, can subsequently increase the precipitation of γ’ [36], which has a strengthening effect on the alloy at elevated temperatures [33]. Therefore, the HSHT temperature coupled with fully solutionised γ’ forming elements contribute to the strength of HSHT PB-DLD variants. 

The dependence of displacement rate was prominent at elevated temperature, particularly when compared to RT, with an increased displacement rate resulting in increased SP peak loads. This corresponds well with literature, where in lower temperature tensile testing, strain hardening is commonly found across a large range of strain rates in nickel superalloys, whereas at higher temperature, a hardening effect is only active as the strain rate is increased, suggesting that strain rate hardening is the primary cause for the increased displacement rate sensitivity [20]. Increasing the temperature of the deformation in an alloy increases the ability for dislocations to move out of their slip planes via cross-slip and climb, increasing the rate of dislocation annihilation [37]. As a time-dependent mechanism, this effect is further pronounced at lower displacement rates. 

Results from EDS analysis revealed differences in carbide precipitation across the C263 build variants. Carbon is introduced into the alloy composition to encourage the formation of carbides to improve mechanical performance. It is largely accepted that carbide precipitates enhance creep properties by pinning grain-boundaries, preventing grain-boundary sliding and hence increasing rupture strength [16]. The extent to which they have a strengthening effect depends on the carbide type, size and morphology [38]. It is not clear from SPT results how the carbide formation directly influences the mechanical rankings of each material variant. A fine, regular dispersion coupled with larger blocky types of what is thought to be M_6_C and MC carbides can be found within intergranular regions within the cast material variant, a feature not present within the PB-DLD microstructures. However, the cast material was not the strongest performer at 780 °C, suggesting that the dissimilarities in carbide formation across variants does not have a significant impact on the SP mechanical response. The large carbides agglomerate on triple point boundaries in the cast material and may act as rupture sites in this instance. Nonetheless, further research is required to confirm carbide type, morphology and critical precipitate sizes [38]. 

### 4.3. Comparison with Uniaxial Testing

Equations have been determined based upon known uniaxial tensile data in order to calculate the yield and UTS for nickel superalloy C263 through SP testing. These equations have helped to highlight the large differences in UTS performance at elevated temperature, with apparent UTS values debited by almost 60% in the SHT condition, whereas the cast C263 is found to retain most its strength at 780 °C. Importantly, the coefficients that have been established for the correlations appear to be closely matched to those that have been calculated in previous research for other metallic material types [22,23,24]. Nonetheless, these differences between the materials highlight the necessity for a more holistic approach to correlate SP to uniaxial test results and further expand the benefits of the miniaturised technique.

## 5. Conclusions

The Small Punch Tensile test method has been successfully applied to build variants of PB-DLD and cast C263 to rank the mechanical performance at both room temperature and 780 °C. The research has also subsequently addressed the effects of build orientation and post process heat treatments on the mechanical response under a biaxial loading condition. The results and analysis from Small Punch testing, microstructural and EDS studies have been used to draw the following conclusions:

1. PB-DLD material exhibited a stronger response to biaxial deformation at RT than traditional cast material. In the SHT variants, this increase in strength is directly attributed to the higher grain boundary density found in these materials in comparison to the cast variant.
2. HSHT PB-DLD variants ranked highest at 780 °C during SPT testing. The dominant cracking form was intergranular across all C263 variations, highlighting the influence of grain size. Elevated temperature testing also revealed a columnar dendritic substructure in SHT builds, which is believed to have had an adverse effect on mechanical performance, and this feature was largely alleviated when the HSHT was applied.
3. Based upon strong correlations of known uniaxial to SP data, equations have been established to correlate SP to uniaxial results for nickel superalloy C263, $\sigma_{y}=\;0.3702\frac{F_{e}}{h_{0}^{2}}$ and $\sigma_{UTS}=\;0.0899\frac{F_{m}}{h_{0}^{2}}+205.2$.
4. From EDS results, it was found that the SHT PB-DLD variant microstructures contained segregates of γ’-forming elements, suggesting that they are not contributing to the precipitation of these phases. The fact that this is not present in HSHT builds suggests that the formation of γ’ is further encouraged in HSHT variants due to the higher solution temperature, strengthening the material during room and high temperature testing.

## Figures and Tables

**Table 1:** Nominal alloy composition of C263 (wt %) [16].

Element	Ni	Co	Cr	Mo	Al	Ti	C	B	Zr
**C263**	Balance	20.0	20.0	5.9	0.5	2.1	0.06	0.001	0.02

**Table 2:** Summary of C263 data for SPT and uniaxial test types for both RT and 780 °C.

C263 Type	Temp. (°C)	UTS (MPa)	Max Load, *F_m_* (kN)	Effective UTS (MPa)	Yield (MPa)	Yield Load, *F_e_* (kN)	Effective Yield (MPa)
Cast	0	649	1.372 ± 0.170	699 ± 62	430	0.335 ± 0.029	495 ± 42
	780	560	0.880 ± 0.129	522 ± 47	338	0.254 ± 0.004	377 ± 5
SHT 90°	0	1268	2.628 ± 0.348	1150 ± 125	843	0.512 ± 0.080	802 ± 118
	780		0.476 ± 0.054	376 ± 19		0.273 ± 0.030	404 ± 44
SHT 0°	0	981	2.429 ± 0.181	1079 ± 65	709	0.463 ± 0.060	686 ± 88
	780		0.742 ± 0.076	472 ± 27		0.309 ± 0.002	457 ± 2
HSHT 90°	0		1.962 ± 0.136	911 ± 49		0.473 ± 0.035	700 ± 51
	780		1.116 ± 0.075	607 ± 27		0.281 ± 0.016	415 ± 33
HSHT 0°	0		2.450 ± 0.322	1086 ± 116		0.416 ± 0.060	616 ± 89
	780		1.008 ± 0.107	567 ± 38		0.280 ± 0.012	397 ± 18
